# A profile of hospital-admitted paediatric burns patients in South Africa

**DOI:** 10.1186/1756-0500-3-165

**Published:** 2010-06-11

**Authors:** Asha Parbhoo, Quinette A Louw, Karen Grimmer-Somers

**Affiliations:** 1 Division of Physiotherapy, Faculty of Health Sciences, Stellenbosch University, PO Box 19063, Tygerberg, 7505, South Africa; 2 Division of Health Sciences, University of South Australia, City East Campus, North Terrace, Adelaide 5001, Australia

## Abstract

**Background:**

Injuries and deaths from burns are a serious, yet preventable health problem globally. This paper describes burns in a cohort of children admitted to the Red Cross Children's Hospital, in Cape Town, South Africa.

This six month retrospective case note review looked at a sample of consecutively admitted patients from the 1 ^st ^April 2007 to the 30 ^th ^September 2007. Information was collected using a project-specific data capture sheet. Descriptive statistics (percentages, medians, means and standard deviations) were calculated, and data was compared between age groups. Spearman's correlation co-efficient was employed to look at the association between the total body surface area and the length of stay in hospital.

**Findings:**

During the study period, 294 children were admitted (f= 115 (39.1%), m= 179 (60.9%)). Hot liquids caused 83.0% of the burns and 36.0% of these occurred in children aged two years or younger. Children over the age of five were equally susceptible to hot liquid burns, but the mechanism differed from that which caused burns in the younger child.

**Conclusion:**

In South Africa, most hospitalised burnt children came from informal settlements where home safety is a low priority. Black babies and toddlers are most at risk for sustaining severe burns when their environment is disorganized with respect to safety. Burns injuries can be prevented by improving the home environment and socio-economic living conditions through the health, social welfare, education and housing departments.

## Background

Injuries and deaths from burns are a serious, yet preventable health problem globally. Every year more than 300 000 people die from injuries due to fires alone [1]. Uncounted numbers are permanently scarred by injuries from hot liquids, electricity and chemicals [1]. Burns in children are reported to be amongst the most prevalent traumatic injuries around the world [2]. In low income countries, and vulnerable populations, burn injuries are reported to be the third most common cause of death in children aged 5 to 14 years, with road traffic injuries and drowning being first and second respectively [3]. According to a recently published article in "Burns", the global incidence of hospitalized paediatric burn patients is unknown [4]. Extrapolations from population-based studies allow global figures to be estimated. The "best guess" figure on the available evidence of total number of hospital burn patients admissions in 2004 was a total of 505 276 [4].

Burns can be devastating injuries for children, the immediate effect of which is compounded by ongoing pain, cosmetic and physical disfigurement, impairment, multiple dressing changes and surgical procedures [5]. The ongoing emotional and psychological impact on the child is often shared by the caregiver or parent [5]. Given that burns are preventable, intervening in the causes of children's burns is essential not only to minimise immediate pain, suffering and health care costs in this vulnerable group, but also to reduce ongoing trauma and disability which could affect children into their adulthood [6].

In middle and high-income countries, considerable progress has been made in lowering rates of burn death and injury for children by efficacious prevention efforts, such as installation of smoke detectors, regulation of hot water heater temperature and producing flame-resistant children's sleepwear [7]. However, most of these advances in prevention and care have been minimally applied in low-income countries, where over 95.0% of burn deaths occur [1]. Fire-related burns mortality rates in children are especially high in South-East Asia (11.6 deaths per 100 000 population per year), the Eastern Mediterranean (6.4 deaths per 100 000 population per year) and Africa (6.1 deaths per 100 000 population per year) [1]. These are significantly higher than the overall 1.0 deaths per 100 000 population per year of similar deaths in high-income countries [1].

South Africa is a generally middle-income, emerging economy with an abundant supply of natural resources along with well-developed financial, legal, communications and energy sectors [8]. Despite being the most developed country in Africa, it is still considered a developing country in terms of health and education standards [8]. In addition, within South Africa, there are densely populated informal settlements, which are high-risk areas for injuries resulting in burns. With electricity largely unavailable to these township inhabitants, lighting is supplied by kerosene lamps and candles, heating is by fires, and cooking occurs on fires and primus stoves situated on the floor [9]. These informal dwellings are sometimes only six square metres in size, posing significant challenges in planning safe kitchen and sleeping spaces [10].

Despite the large amount of international literature on paediatric burns and their causes, there is limited information on the profile of paediatric burns in South Africa. This paper describes burns in a cohort of children admitted to the main South African paediatric burns hospital over a six month period in 2007.

## Patients and methods

***Ethical considerations***: Ethical approval was obtained from the Committee of Human Research at Stellenbosch University, the School of Child and Adolescent Health at the Red Cross Children's Hospital (RCCH) as well as the University of Cape Town.

***Study design***: Retrospective case note review.

***Study setting***: The Red Cross Children's Hospital (RCCH) in Cape Town, South Africa, is a 51 year old tertiary academic hospital which admits patients up to 13 years of age [10]. It is the only paediatric hospital in Sub-Sahara Africa with a trauma unit and a burns unit dedicated exclusively to children. It thus provides care to the majority of children requiring hospitalisation for burns in the Western and Eastern Cape. These two areas encompass an area of 300 000 square kilometres and a combined population of approximately twelve million people [11]. The burns unit, where the study was conducted, is a seventeen-bed ward where approximately 900 patients are admitted each year. The criteria for admission to the unit are that the patient has to have a burn of more than ten percent total body surface area (TBSA). However all burn injuries involving an inhalational component, electrical injuries, burns to face, hands, or the perineum, or circumferential burns will also be admitted, even if the TBSA is less than 10.0% [12].

***Study population***: Children in sub-Sahara Africa, and specifically within the Western Cape of South Africa.

***Sample***: The study sample included all consecutively-admitted patients to the Red Cross Children's Hospital from the 1 ^st ^April 2007 to the 30 ^th ^September 2007.

### Instrumentation

A project-specific data capture sheet was designed by the authors, assisted by two clinician experts in the burn units at RCCH (a surgeon and a physiotherapist). The content was based on a review of published burns profile studies [5,13-16]. The data capture sheet included information of patient age and gender, the date of birth, injury, admission and discharge. Cause of burn was recorded using four categories (hot liquids, fire, electrical, and chemical). Area of the burn was recorded in categories of body parts i.e. right or left hands, both hands, trunk etc). Depth of burn (partial and full thickness, or superficial categories) and percent of total body surface area affected (TBSA) were recorded. Details of the actual mechanism of the burn were also recorded so that each burn could be further investigated for the purpose of assisting with preventative measures. Mechanisms of hot liquid burns were divided into kettles being pulled over by their hanging cords (in homes where electricity was available), pots and cups of hot liquid being knocked over, and bath water being too hot. Mechanisms of fire burns were subcategorized into flash burns and burns caused by the "shack" catching alight. "Shacks" are informal dwellings, typically found in informal settlements, where there are no proper roads, electricity or sanitation. They are constructed of sheet metal, wood, corrugated cardboard and scrap material. The "shack" is often assembled from a combination of all or some of these materials. Ethnic groups were categorized as Black, White, Coloured and Indian as these are the four main racial classifications in South Africa. A "coloured" person is a person who has white and one or many other racial backgrounds and is not considered to be a derogatory term. It is an acceptable term to use in South Africa for statistical purposes. The data collection form is provided in Appendix 1.

### Procedures for data collection

#### Pilot study

Prior to this pilot study, the primary researcher (PR) showed the research assistant (RA) how data should be captured from the medical records onto the data capture sheet. The aim of a pilot study was to determine if the RA was efficient in collecting the required data and to establish if there was consistency between the data collected by the RA and PR. Discrepancies in the data collected would indicate that the RA required further training. During the pilot study, the same information of 20 patients was independently collected from the medical records by the PR and the RA. This information was gathered during an afternoon over a three hour period. When the RA and the PR compared the information gained from the folders, a 100% agreement on all items collected was reached between them.

#### Data collection

Children eligible for inclusion in this study were identified from the list of admissions to the burns unit between the 1 ^st ^April 2007 and the 30 ^th ^September 2007. These patients' medical records were requested from the medical records department at RCCH. Data was extracted by the same two researchers (the PR and RA, both physiotherapists). They used the same protocols to extract and document data on the data collection sheet [additional file 1]. The data for this study was collected by the RA in the same manner that information was collected in the pilot study. All data was captured in a period of four mornings over a two week period. Validation checks were in place throughout data collection to ensure accuracy of data extraction. The PR checked every 10 ^th ^data collection form to determine if the data collected was precise and complete. She obtained the medical record corresponding to the data captured by the RA and checked to see that the data collected was accurate and complete. The RA was unaware that every 10 ^th ^file was checked for accuracy.

***Data Handling/data management***: Data was entered and stored in a purpose built MS Excel sheet.

***Data analysis***: Analysis was undertaken using MSExcel functions, describing the data by percentages, central tendency (medians, means) and measures of variability (such as standard deviation). Spearman's correlation co-efficient was employed to estimate the association between the total body surface area (TBSA) and the length of stay in hospital. This statistical test was used to account for abnormally distributed data. Differences between equal interval data were tested using Student T-tests.

## Findings

The sample consisted of 294 children (115 (39.1%) girls and 179 boys (61.9%), with no deaths having occurred during the study period. The mean age for boys was 2.7 years (SD 2.5 years), ranging from 0.4 years to 12.7 years. The mean age for girls was 3.0 years (SD 2.5 years), ranging from 0.4 years to 12.5 years. There was no significant difference in age between girls and boys (*p > 0.05*). Most of the patients (89.0%) admitted were from informal settlements and lived in "shacks", with the majority of them not having electricity (71.0%), whilst 11.0% lived in a brick house with or without electricity. Only 7.0% of the total population living in "shacks" had electricity.

### Cause of burns

The majority of children (244 (83.0%)), were burnt by hot liquids (Table 1). This included hot beverages being spilt (25.0%), boiled water spilt from a pot (8.0%), hot liquid from a kettle being spilt (48.0%), accidently placing a child in boiling bath water (1.7%) and hot oil splashes (1.0%). In contrast, 44 (15.0%) children were burnt with fire, of which 70.0% of those burns were due to primus stoves being knocked over. Five (1.7%) children were burnt by exposed electrical wires, There were no gender differences in the causes of burns, or in the age at which the different caused burns occurred *(p > 0.05)*. This is illustrated in Table 1.

**Table 1:** Distribution of study population by gender and cause of burn (n = 294)

	HOT LIQUID	FIRE	ELECTRICAL		CHEMICAL
**FEMALES**	97	17	1		0
	(33.0%)	(5.7%)	(0.3%)		(0.3%)
Mean age (yrs)	2.9	3.7	8.9		N/A
SD (years)	2.4	2.6	N/A		N/A
Range (years)	0.4 - 12.5	0.9 - 8.9	N/A		N/A
					
**MALES**	147	27	4		1
	(50.0%)	(9.0%),	(1.0%)		(0.3%)
Mean age (yrs)	2.4	4.7	4.6		1.7
SD (years)	2.2	3.1	0.9		N/A
Range (years)	0.4 - 12.7	0.7 - 10.8	2.5 - 4.5		N/A

**Total**	**244**	**44**	**5**		**1**

### Cause of burn related to ethnicity

Of the hot liquid burns, 142 (58.0%) were from the black population, 139 (48.0%) of the total population who encountered these burns were aged two years or younger, and 61 (21.0%) of these children were aged under one year. 80.0% of the electrical burns occurred in the black population and were due to exposure of live electrical wires. No Indian children were admitted during the study period. The ethnic distribution of burn types is reported in Table2.

**Table 2:** Distribution of study population by ethnic group and cause of burn (n = 294)

ETHNIC GROUP	HOT LIQUID	FIRE	CHEMICAL	ELECTRICAL		TOTALS
Black	142	26	1	4		173
Coloured	99	18	0	1		118
White	3	0	0	0		3
Indian	0	0	0	0		0
**TOTALS**	**244**	**44**	**1**	**5**		**294**

### Causes of burn within age groups

Table 3 presents the different age categories of the study population and the causes of their burns. Overall, the children under the age of three represented the largest group of children burnt, with 78 (26.5%) aged between one and two years, as reported in Table 3.

**Table 3:** Distribution of study population by age category and cause of burn (n = 279)

AGE CATEGORY	HOT LIQUID	FIRE
**(0-1]**	61 (20.7%)	3 (0.6%)
**(1-2]**	78 (26.5%)	9 (3.0%)
**(2-3]**	35 (11.9%)	4 (1.3%)
**(3-4]**	17 (6.5%)	6 (2.0%)
**(4-5]**	14 (4.7%)	5 (1.7%)
**(5-6]**	11 (3.7%)	3 (1.0%)
**(6-7]**	10 (3.4%)	2 (0.6%)
**(7-8]**	6 (2.0%)	3 (1.0%)
**(8-9]**	3 (1.0%)	2 (0.6%)
**(9-10]**	2 (0.7%)	2 (0.6%)
**(10-11]**	1 (0.3%)	2 (0.6%)

**TOTALS**	**238**	**41**

The square bracket on the right denotes the age up until the number, but excluding the number itself, ie: (0-1] includes all ages from 0 years to 1 year, but excludes 1 year itself. The burns caused by chemical and electrical means have been excluded as the numbers are insignificant.

### Depth of Burn

Most of the children (71.4%) sustained deep partial thickness burns (Table 4). The children who sustained full thickness burns (11.6%), were mainly burnt by hot water from kettles. Fire burns, specifically due to flash fires and children's clothing catching alight, were responsible for 25.0% of the full thickness burns. "Superficial" in Table 4 includes the superficial and superficial partial categories.

**Table 4:** Distribution of study population by depth of burn (n = 294)

DEPTH OF BURN	% POPULATION
Superficial	50 (17.0%)
Deep partial	210 (71.4%)
Full thickness	34 (11.6%)

**TOTALS**	**294**

### Percentage of burn (TBSA)

Most of the patients sustained burns of 20.0% or less of TBSA and 69.0% of this category was aged three years or younger. Children aged 1-2 years appeared to be most at risk of extensive burns. During the study period, only one child sustained a burn of more than 60.0% as demonstrated in Table 5.

**Table 5:** Distribution of study population by age category and average TBSA (n = 294)

AGE CATEGORY	n	0.5-20%	21%-40%	41%-60%
(0-1]	*64*	62	2	0
(1-2]	*92*	89	2	1
(2-3]	*42*	37	3	2
(3-4]	*24*	21	3	0
(4-5]	*21*	20	0	1
(5-6]	*14*	11	3	0
(6-7]	*12*	11	1	0
(7-8]	*9*	7	2	0
(8-9]	*6*	6	0	0
(9-10]	*4*	3	1	0
(10-11]	*3*	1	2	0
(11-12]	*1*	1	0	0
(12-13]	*2*	2	0	0
**TOTAL**	**294**	**271**	**19**	**4**

The square bracket on the right denotes the age up until the number, but excluding the number itself, ie: (0-1] includes all ages from 0 years to 1 year, but excludes 1 year itself.

### Body areas burnt

**Figure 1:**
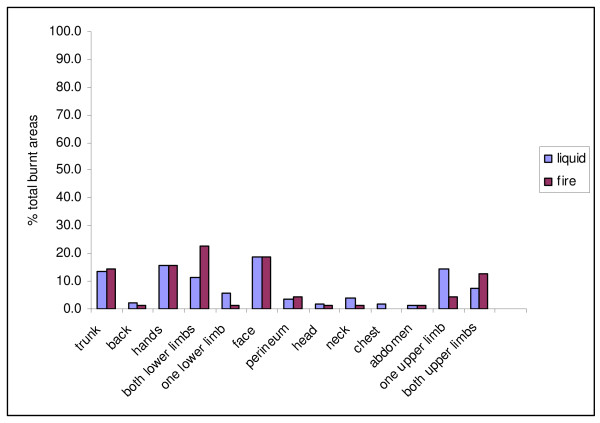
% total body areas burnt by the most common causes of burn (n = 533)

There was a total of 533 burnt areas in the sample. Overall, the greatest number of body areas burnt was six (mean 1.8 (SD 1.0)). We considered the differences in body areas burnt by liquid and the fire, by expressing the number of times that each body area was burnt, as a percentage of the total number of body areas burnt, by each mechanism of burn. There were 449 body areas burnt by hot liquid, and 70 body areas burnt by fire. The comparative frequency of body area burns is presented in Figure 1.

Considering the different causes of burns, the children burnt by hot liquid burnt on average 1.8 body areas (SD 1.0) (range 1-6), and the children burnt by fire, burnt on average 1.7 body areas (SD 0.9), range 1-4. All electrical burns were on the hands (n = 5), and were caused when children touched exposed electrical wires that were lying around. There were too few children with the other causes of burns to make meaningful comparisons. The mean body area burnt was 10.0% (SD 8.9) ranging from 0.5% to 62.0%. Considering the percentage of total body area burnt, the cause of burn exerted no significant effect.

### Length of Stay in Hospital

Table 6 reports only moderate correlation between the length of stay of the patient in hospital and the TBSA (Spearman r = 0.36). This was not influenced by location of the burn, or its depth.

**Table 6:** Distribution of the study population by TBSA and length of stay in hospital (n = 294)

LENGTH OF STAY	(0%-10%)	(11%-20%)	(21%-30%)	(31%-62%)
**Mean**	15.17	34.86	10.15	71.50
**SD**	30.50	46.13	12.04	86.18
**Range (days)**	0-381	1-265	1-31	6-214
**Median**	8.00	22.00	23.00	24.50

## Discussion

This paper provides evidence for the global picture of the high-burns-risk environments in which some children in developing countries live. The Western Cape informal settlements were the source of most of the burns, in which the unsafe nature of home heating, lighting and cooking was highlighted. This paper exemplifies the need for preventative measures to be put in place and which could be addressed by better home design, and by educating parents about safe home environments, and child supervision.

Burn injury incidence was the highest amongst toddlers and the second highest amongst babies aged younger than one year. Our findings were congruent with epidemiological studies from India, France and other parts of Africa [5,14,17,18]. This highlights the high incidence of burns in very small and vulnerable children, who are burnt by mechanisms directly relevant to unregulated living environments in a developing country. A recent review, looking at different socio-economic nations (for instance USA versus India), found that issues related to the causes and prevention of paediatric burns were similar across nations. The importance of providing children with a safe home was a common trend, as was the importance of educating parents and caregivers in recognizing and addressing risks for burns, and reinforcing the importance of their role in ensuring the safety of their child [19]. It appears that paediatric burn prevention strategies have the potential to be standardized across developed and developing countries, although they would require different mechanisms of implementation to address different socio-economic status, education, literacy and opportunity to change [20].

In the current study, hot water was responsible for the majority of injuries. This was also reported by the World Health Organization (WHO) in their 2008 global report, which indicated that the only difference was in the mechanism of the burn, when comparing first and third world countries. The findings in this study also concur with studies performed in developed nations [21-23]. There were no differences between developed and developing countries in terms of causes of burns, and burn prevention strategies. The most common cause of paediatric burns, independent of country and socio-economic status, was contact with hot liquids, resulting in scalds. Flames were the second most common cause of burns. However, the environmental circumstances of scalding differed between developed and developing countries. In developed countries, with formal housing and electricity supplies, scalds were mostly caused by the child pulling at kettle cords. In developing countries, where overcrowded informal housing settlements and lack of access to utilities predominates, scalding occurred when a pot or vessel of boiling liquid on a fire, or gas stove at ground level, was knocked over [23].

In this study, the mechanism of the hot water burns was tipping the container over. Many of the burns described in this paper could have been prevented if hot water containers, or stoves are placed at a higher level, out of reach of children, or if bath water was tested first. It is of concern that face and hand burns were amongst the most common areas burnt in this manner. Facial and hand burns could result in short and long term functional and psychological impairment, and many will require cosmetic surgery to improve scarring even if the burn itself has not caused any loss of function.

In the black South African community, children from birth to two years are normally carried on the mothers' back rendering them completely immobile. However, older children are independently mobile, and are not always under the guiding eye of a parent. Toddlers who are learning to walk and investigating their environment are naturally unstable due to their ambulatory development. They may be prone to grabbing kettle cords and tablecloths to steady themselves, which could explain why children in this category were burnt more frequently than any others [24,20].

With the older age groups in this study, the burns were more commonly related to 'shack' fires, where the entire dwelling burnt down, or children's clothing caught alight. A small number of fire burns were caused by children throwing spirits or turpentine onto an already burning fire, causing the fire to "flame out". These flash burns are of real concern since they caused 25.0% of the full thickness burns and occurred when children's clothing caught alight. There appeared to be no security for fire-provoking substances such as chemicals or matches, putting them in easy reach of children. The predominant reason for electrical burns was exposed, live, non-insulated electrical wiring. In recent years in the informal townships in South Africa, there has been a spate of theft of electrical wire as well as individuals trying to "pilfer" electricity by illegally tapping into the government electricity cables. This leaves unsheathed electrical cabling exposed to the hands of children [23]. Whilst they may only affect a small surface area, these electrical burns often produce deep burns which involve tendons and joints. Despite corrective surgery, these burns often result in impaired hand function with permanent consequences.

There was no significant gender difference for burns occurring in children aged younger than three years. This indicates that both genders are equally exposed to the same dangers for sustaining hot water and fire burns. In the older group aged four to nine years old, boys were burnt more with fire, and girls with hot liquid. Boys were more likely to gain access to substances that caused "flare outs" of existing fires, whilst girls were more likely to be assisting with household chores of cooking and cleaning, and therefore were potentially more exposed to pots of hot liquid.

A potentially longer period of exposure to the cause of burn results in a potentially longer contact time with the skin, resulting in deeper burns. Many of the severe burns resulted from hot water that had just boiled and had spilled over the child. Since most of the children burnt in this manner were under three years of age, it is possible that they were not able to able to remove their drenched clothing as quickly as an older child. This would have resulted in the hot water having a longer contact time with the skin and producing a more significant burn.

There was no correlation between the length of stay and the TBSA burnt. This finding highlighted that patients with small percent of TBSA burnt had a longer stay in hospital than patients who had burns greater than 20.0%. This was due to recurrent wound infections, or small but deeper burns which took longer to heal, or required repeated surgery.

A potential limitation of this study is that it only reports on children admitted for burns to one major tertiary hospital. It is plausible that other burnt children in the Western Cape were not accounted for in this study, as they may have been admitted to private hospitals. However given the lack of private health insurance by many South Africans, as well as the world-renowned burns management at the RCCH, which offers free treatment for children under the age of six years old, the number of burns children not accounted for in this study is estimated to be small. Moreover this study did not capture information on children with burns to less than 10.0% of their bodies (unless a smaller burn was a criterion for admission to hospital). The outpatient unit at the RCCH services between 1300-1600 patients in their bi-weekly outpatient clinics, per year. Many of these patients would not have been admitted to the burns unit. Factors such as smoke inhalation and co-morbidities were also not accounted for. This is a shortcoming of this study. Lastly, the data was collected during a six month period and it is possible that the information obtained could be specific to the seasons it was collected in.

## Conclusion

The most commonly burnt children admitted to hospital in the Western Cape, South Africa, were predominantly black (this is representative of the total black population of the Western Cape which is 60.2%) [25]. The main causes of burn injuries were hot water from tipping containers above the child's head, or at ground level, and unattended fires. These causes of burns mirror those of other developing countries for burns in children of this age. In South Africa, most hospitalised burnt children came from informal settlements where home safety is a low priority. Burns injuries could be prevented by improving the home environment and socio-economic living conditions through the health, social welfare, education and housing departments.

## Competing interests

The authors declare that they have no competing interests.

## Authors' contributions

All authors have made substantial contributions to all the following:

AP, QAL and KGS were involved in the conception and design of the study, or acquisition of data, or analysing and interpretation of data. AP, QAL and KGS were involved in drafting the article or revising it critically for important intellectual content. AP, QAL and KGS were involved in the final approval of the version to be submitted.

## Supplementary Material

Additional file 1 **Profile of the burns patient from 01 April 2007 to 30 September 2007**. this file contains the details of the patient's demographic information to be collected from the patient's medical records. Click here for file
