# Supplementary material for: A profile of hospital-admitted paediatric burns patients in South Africa
Source: BMC Res Notes. 2010 Jun 11;3:165. doi: 10.1186/1756-0500-3-165 (PMC2893535; doi:10.1186/1756-0500-3-165)
Supplement: Additional file 1 — Profile of the burns patient from 01 April 2007 to 30 September 2007. this file contains the details of the patient's demographic information to be collected from the patient's medical records. [file 1756-0500-3-165-S1.PDF]

Additional file 1

Title: Profile of the burns patient from 01 April 2007 to 30 September 2007

Description: this file contains the details of the patient’s demographic information to be collected from the patient’s medical records.

Patient details

1. STUDY NUMBER ……………………..

2. Folder number

3. Gender: Male -0 / Female-1

4. Date of Birth _ _ _ _ _ _ (YYMMDD)

5. Age at admission _ _ _ (months)

6. Ethnic group:
   - -Black 0
   - -White 1
   - -Coloured 2
   - -Indian 3
   - -Other 4 (Please specify) ____________________ ☐

7. Health district:
   - -Western Cape (Northern) 0
   - -Western Cape (Southern) 1
   - -Western Cape (Eastern) 2
   - -Western Cape (Western) 3
   - -West coast / Winelands 4
   - -Boland/ Overberg 5
   - -Karoo 6
   - -Other 7 (Please specify) ____________________ ☐

8. Type of dwelling: Brick house with electricity 1
Brick house without electricity 2
Shack with electricity 3
Shack without electricity 4
Other 5
(Pleases specify) _______________

Details of burn injury

9. Date of burn _ _ _ _ _ _ (YYMMDD)

10. Date of admission _ _ _ _ _ _ (YYMMDD)

11. Number of days between burn injury and admission

12. Date of discharge/death _ _ _ _ _ _ (YYMMDD)

12. Total days spent in hospital _ _ _ (days)

14. Cause of burn: Hot liquid or substance 0
Fire 1
Electrical 2
Chemical 3
Abrasion 4

Please specify the mechanism of the injury

----

15. TBSA _ _ _ %

16. Areas burnt: Face 0
Right hand 1
Left hand 2
Both hands 3
Trunk 4
Right Upper limb 5
Left upper limb 6
One or both lower limbs 7
Face and one/both hands 8
Face and one/both upper limbs 9
Other 10

(Please specify what the "other" area was and what the "multiple" areas if there were) ___-
____

17. Depth of burn: Superficial 0
Partial 1
Full thickness 2
